# Sarcoidosis as an Uncommon Cause of Chest Pain: A Case Report

**DOI:** 10.7759/cureus.77913

**Published:** 2025-01-24

**Authors:** Elisabete Ribeiro, Letícia Marques Leite, Isabel Bessa, João Pacheco, Filipa Gonçalves

**Affiliations:** 1 Internal Medicine, Hospital Senhora da Oliveira, Guimarães, PRT; 2 Pathology, Hospital Senhora da Oliveira, Guimarães, PRT

**Keywords:** adenopathies, chest pain, corticotherapy, non-caseating granulomas, sarcoidosis

## Abstract

Sarcoidosis is a multisystem granulomatous disease of unknown etiology. Despite primarily affecting the lung, sarcoidosis can affect any organ, resulting in various clinical manifestations.

We present a case of a 56-year-old man who developed thoracic pain over several months along with skin lesions. The chest CT revealed multiple mediastinal lymphadenopathies. The patient underwent an endobronchial ultrasound, and a lymph node biopsy was performed. The histological results showed lymphoid cells and small epithelioid granulomas, while bronchoalveolar lavage revealed lymphocytosis, with a significantly elevated CD4+/CD8+ ratio. Based on the results, a diagnosis of sarcoidosis was presumed. The study was concluded with cardiac MRI due to complaints of chest pain, which also confirmed cardiac involvement. The patient was successfully treated with corticosteroids, exhibiting significant improvements and recovering completely during the follow-up period.

Despite cardiac involvement in sarcoidosis being rare, we present this case to emphasize the challenges in diagnosis, requiring high clinical suspicion and the use of complementary imaging methods, such as cardiac magnetic resonance. We also emphasize the importance of early initiation of corticosteroid therapy to prevent major complications and promote recovery.

## Introduction

Sarcoidosis is a systemic inflammatory disease of unknown etiology, with the formation of granulomas in many organs [1]. In approximately 90% of cases, the pulmonary system is affected [1,2]. However, this is a multi-organ disease that may involve lymph nodes, the skin, the heart, the liver, and the central nervous system, resulting in a wide spectrum of clinical presentations [2,3]. Cardiac involvement is rare, and many patients with pulmonary and systemic sarcoidosis have clinically silent cardiac involvement. Occurs in 3-10% of patients, but certain autopsy reports and imaging studies have shown a higher prevalence of cardiac involvement [4,5]. The patient may be asymptomatic or may present with mild symptoms, such as chest pain and shortness of breath, or life-threatening, such as cardiac arrhythmias, congestive heart failure, or sudden cardiac death [4,5]. Although sarcoidosis usually has a benign course, the presence of clinical cardiac involvement has traditionally been linked to a poor outcome if therapy is delayed [2,4,5].

## Case presentation

A 56-year-old male with a medical history of hypertension and insulin-treated type 2 diabetes mellitus presented to the emergency department (ED) with chest pain radiating to the right scapula and dyspnea for several months and cough. The pain was unrelated to exertion and respiratory movements. The patient underwent a chest X-ray, which revealed diffuse, bilateral, and non-specific pulmonary infiltrate; a CT scan was performed for clarification. The chest CT revealed multiple mediastinal lymphadenopathies. The patient was prescribed amoxicillin and clavulanic acid for a respiratory infection, along with an anti-inflammatory medication, and demonstrated clinical improvement. Several months after this episode, the patient returned to the ED with the same symptoms: more intense chest pain in the right hemithorax, radiating to the right scapula, and dyspnea, without fever. Additionally, the patient reported the appearance of non-pruritic skin lesions on the elbows and knees within eight months of evolution. The patient denied asthenia, anorexia, night sweats, or weight loss.

**Figure 1:**
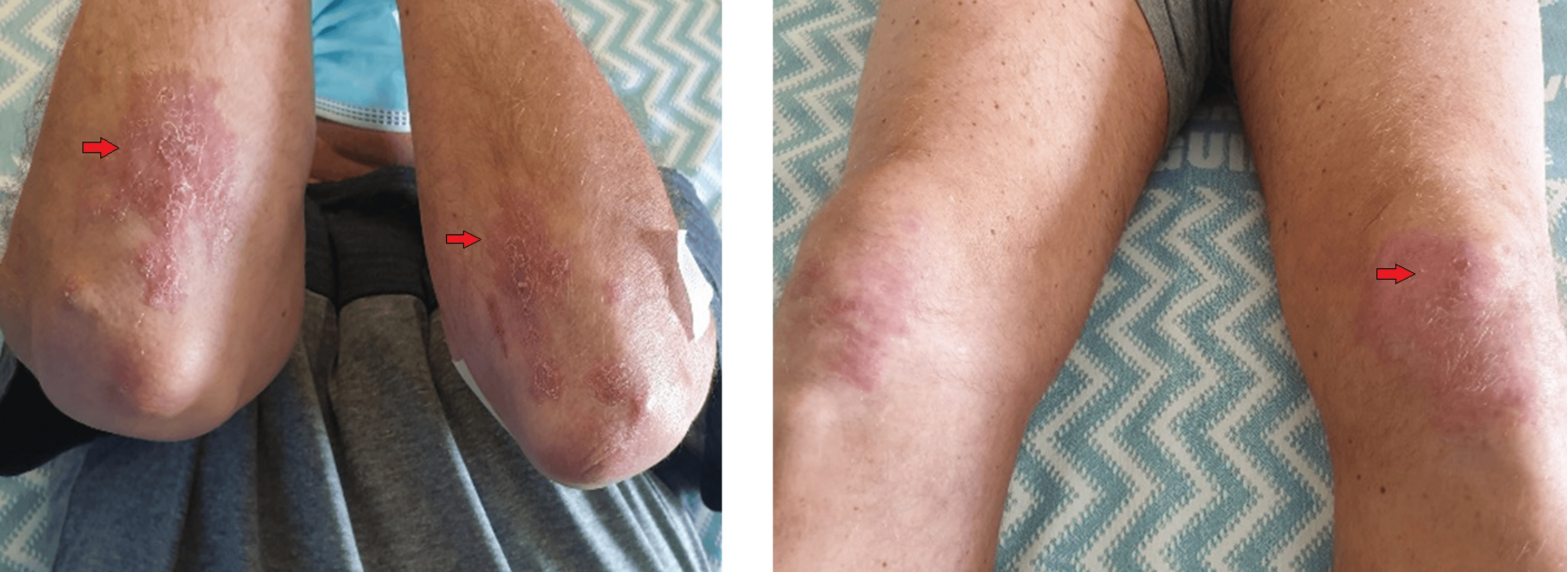
Erythematous lesions on the extensor surfaces of the elbows and knees

The patient had no significant family history, no relevant occupational exposure, and no history of toxic habits. Upon evaluation in the ED, the patient was hemodynamically stable, afebrile, and eupneic, with oxygen saturation (SaO_2_) at 98%. Physical examination revealed slightly scaly erythematous lesions on the extensor surfaces of the elbows and knees (Figure 1), with no other notable abnormalities.

Laboratory tests revealed a mild elevation in transaminase levels (less than three times the upper limit of normal) and a slight increase in markers of myocardial necrosis, as shown in Table 1.

**Table 1:** Laboratory findings of myocardial necrosis and systemic inflammatory markers upon admission to the ED and discharge PBNP: B-type natriuretic peptide; CK mass: creatine kinase mass; TGO: aspartate transaminase; TGP: alanine transaminase; CRP: C-reactive protein; ESR: erythrocyte sedimentation rate

	Admission	Discharge	Normal range
High-sensitivity troponin (ng/L)	385 (0 hour), 878 (2 hours)	20	<45
Myoglobin (ng(mL)	138	98	14-106
PBNP (pg/mL)	153	153	<12
CK mass (ng/mL)	11.46	7	<7.2
TGO (UI/L)	45	35	12-40
TGP (UI/L)	46	37	7-40
CRP (mg/L)	4.7	3	<3
ESR (mm)	11	10	0-12

**Figure 2:**
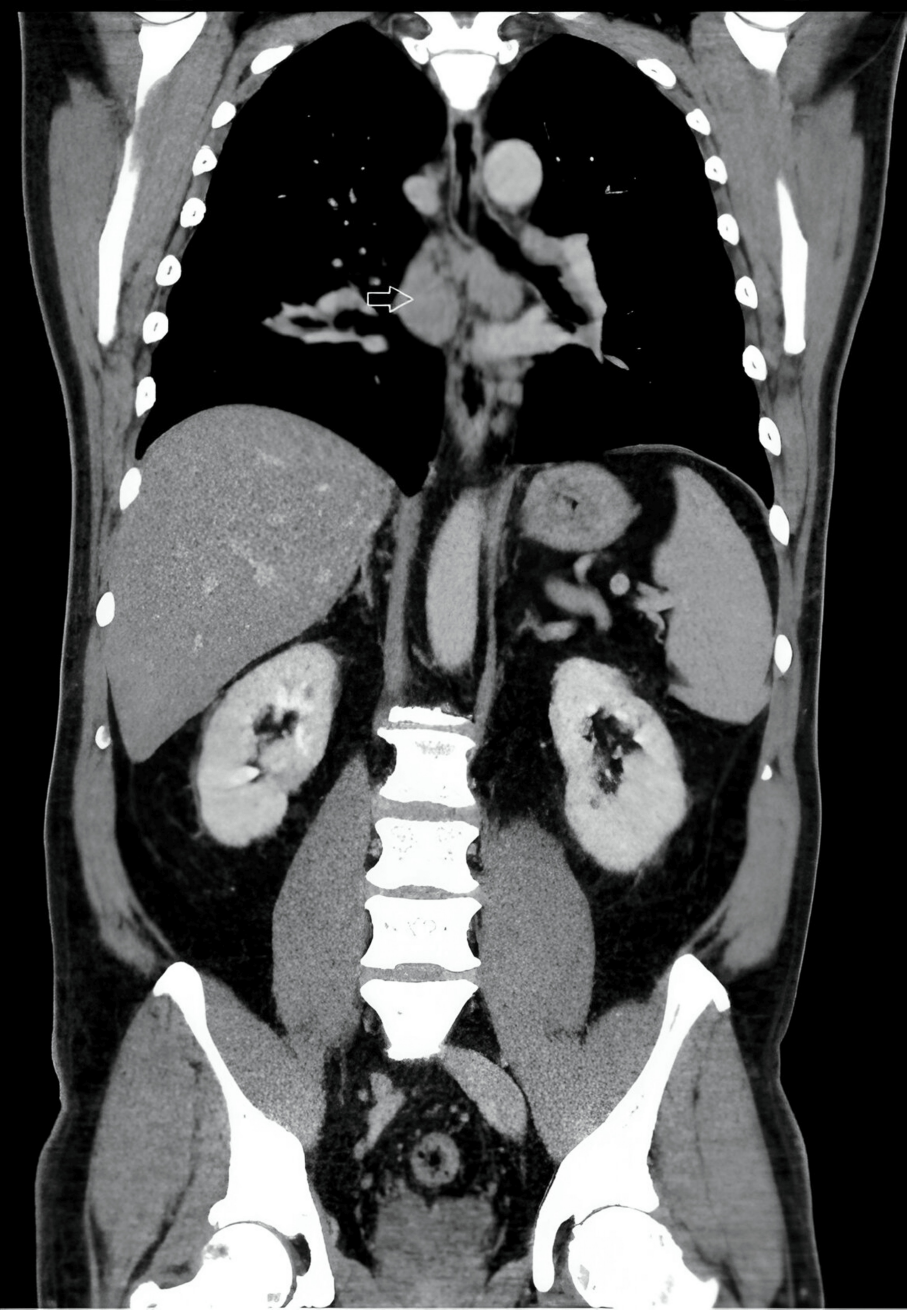
CT scan revealing multiple mediastinal and bilateral hilar adenopathies, the largest measuring approximately 28 mm

The electrocardiogram was in sinus rhythm, with no signs of acute ischemia, no changes in PR segment, or brunch blockages. An echocardiogram demonstrated slight hypertrophy of the left ventricular walls, with normal left ventricular function and no segmental kinetic abnormalities. Arterial blood gas analysis showed no respiratory failure, and a new chest CT was performed to reevaluate the lesions. The scan revealed multiple mediastinal and bilateral hilar adenopathy, with non-calcified nodes and no signs of central necrosis (Figure 2), the largest measuring approximately 28 mm.

The patient was admitted for further investigation.

In the initial days of hospitalization, the patient presented with chest pain without dyspnea or other symptoms. Regarding the adenopathy documented on the CT scan, to rule out lymphoproliferative disease, the patient underwent a bone marrow study that did not reveal immunophenotypic alterations in lymphocytes suggestive of lymphoproliferative disease.

The patient presented negative viral serologies for hepatitis A, B, and C viruses and human immunodeficiency virus; negative syphilis screening; and negative interferon-gamma release assay (IGRA). Immunological studies demonstrated an erythrocyte sedimentation rate of 16 mm (N: 0-12 mm), negative antinuclear antibody (ANA), negative anti-dsDNA, no complement consumption, and normal angiotensin-converting enzyme (ACE) levels (80 U/L, N: 35-90 U/L).

**Figure 3:**
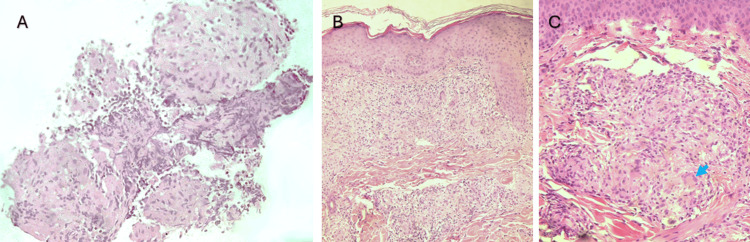
(A) In the EBUS-TBNB of a hilar lymph node, non-necrotizing, well-demarcated epithelioid granulomata surrounded by a lymphocytic infiltrate were observed within fragments of fibrous tissue (HE, 200x). (B) On the skin biopsy, a dermal chronic non-necrotizing granulomatous inflammation is present, predominantly on the superficial dermis (HE, 100x). (C) Skin granulomata showed confluence and comprised some giant multinucleated cells (arrow); a slight lymphocytic infiltrate surrounded the granulomata (HE, 200x). Ziehl-Neelsen and PAS stains were negative for acid-fast bacteria and fungi in both lymph nodes and skin samples (not pictured) EBUS-TBNB: endobronchial ultrasound-guided transbronchial needle aspiration; PAS: Periodic Acid-Schiff

The patient underwent endobronchial ultrasound with lymph node biopsy and bronchoalveolar lavage (BAL). BAL was negative for fungal infections, acid-fast bacilli (AFB), and malignant cells. Histological examination revealed lymphoid cells and small epithelioid granulomas (Figure 3A). A skin biopsy of the lesions revealed chronic granulomatous inflammation with epithelioid granulomas (Figures 3B-3C), negative AFB staining, and no dysplasia or malignancy. BAL cytology revealed lymphocytosis with an elevated CD4+/CD8+ ratio (ratio 9.28), without malignant cells.

**Figure 4:**
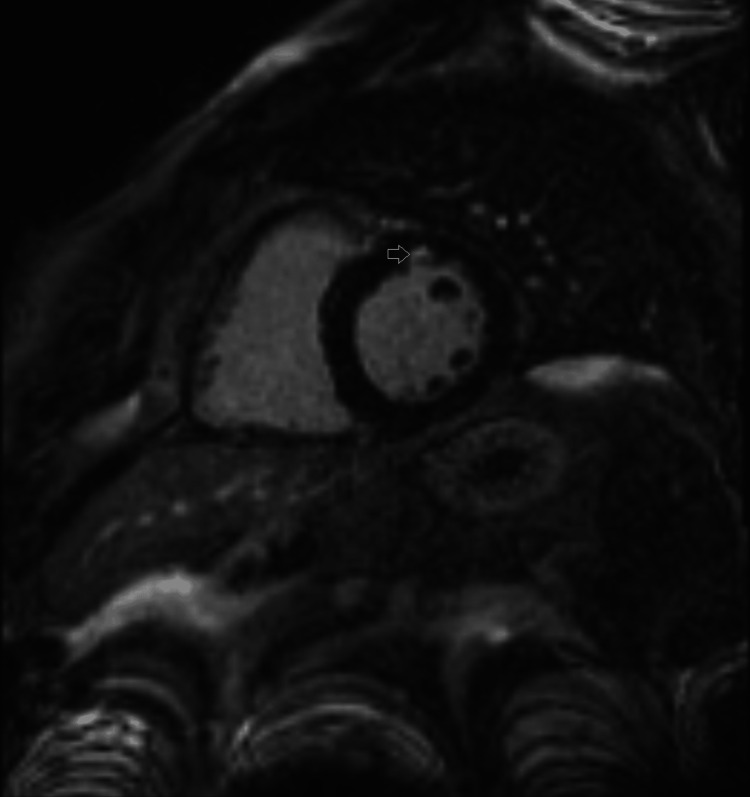
Cardiac MRI showing a focal area of late gadolinium enhancement in the mid-wall (non-ischemic pattern) located at the basal segment of the anterior wall

During hospitalization, due to chest pain and also due to the hypothesis of sarcoidosis, a cardiac MRI was performed. This showed a non-dilated and non-hypertrophic left ventricle with global systolic function at the lower limit of normal (left ventricle ejection fraction (LVEF) 53%), a non-dilated right ventricle with normal global systolic function, and a late gadolinium enhancement focus in a mid-mural location (non-ischemic pattern) located at the level of the basal segment of the anterior wall. However, there was no hyperintensity on T2-weighted images, indicating focal myocardial fibrosis. The inflammatory activity and the other systemic findings were suggestive of myocardial involvement caused by sarcoidosis (Figure 4).

The patient was discharged with a diagnosis of sarcoidosis with pulmonary, cutaneous, and cardiac involvement and initiated therapy with prednisone 30 mg/day, with subsequent re-evaluation. At the one-month, six-month, and one-year follow-ups, the patient presented with normal pulmonary function tests, and the chest CT showed a reduction of the size of mediastinal lymph nodes without pleural-parenchymal disease. Additionally, the skin lesions decreased in size with the treatment. The patient continued to be monitored and was prescribed a daily dosage of 5 mg of prednisone. Throughout this period, the patient exhibited no symptoms, and the echocardiogram and Holter conducted showed no changes.

## Discussion

Sarcoidosis is a systemic granulomatous disease of unknown etiology that affects 15 to 22 individuals per 100,000 inhabitants [6]. It occurs worldwide and can affect people of any age, race, or ethnic group, and both sexes, being more prevalent in females [3,7]. Peak incidence has been reported between the ages of 30 and 50 for males and between 50 and 60 for females [3]. Data suggest that a family history of sarcoidosis increases the risk of developing the disease, with a risk that is 3.7 times higher for first-degree relatives [3].

The exact cause of sarcoidosis remains unknown. Studies suggest that it develops in genetically predisposed individuals following exposure to an environmental trigger. The immune response involves T cells, leading to the formation of non-caseating granulomas, a histological hallmark of this disease [2,3]. The clinical presentation of sarcoidosis is variable and can affect any organ [3]. It depends on multiple factors such as the affected organs, epidemiological factors (age, sex, and ethnicity), and the duration of the disease [2,3]. Since it's a multisystemic disease, there may be nonspecific symptoms such as asthenia, fatigue, or cognitive impairment.

The diagnosis of sarcoidosis is based on suggestive clinical features and the identification of non-caseating granulomas in one or more tissue samples, after ruling out other causes of granulomatous disease [3,7]. It is crucial to exclude other diagnoses that often present with similar clinical manifestations, such as respiratory infections (e.g., tuberculosis) or neoplasms. Patients with pulmonary involvement may present subacute or chronic respiratory symptoms, as described in the case presented. The diagnosis is suspected based on pulmonary function tests, BAL samples, and findings on chest X-ray or CT scan, such as bilateral hilar lymphadenopathy (or unilateral, more commonly on the right side) with a micronodular pattern, which is highly specific for sarcoidosis [5]. However, to establish the diagnosis, biopsy samples from the lungs or mediastinal lymph nodes are necessary [3,5].

In the presented case, the diagnosis of sarcoidosis with pulmonary and cutaneous involvement was confirmed due to imaging findings revealing hilar and mediastinal lymphadenopathies, with granulomatous lesions confirmed in biopsies of the lymph node and one of the skin lesions. Also, lymphocytosis in BAL with a CD4+/CD8+ ratio greater than 3.5 is a finding highly specific that supports the sarcoidosis hypothesis. This case also highlights the prevalence of cutaneous involvement, occurring in 25% of patients and generally being an early finding [1], as demonstrated in this case.

In regards to serum biomarkers, these are not specific for pulmonary involvement, and although approximately 90% of patients with pulmonary sarcoidosis have lymphocytosis in BAL, this finding is not specific for sarcoidosis and can occur in other lung diseases [3,5]. Additionally, the role of the CD4/CD8 T-lymphocyte ratio in BAL remains controversial, although some studies suggest that ratios greater than 3.5 have a specificity greater than 95% for the diagnosis of sarcoidosis [5,8]. ACE levels are a non-specific marker of sarcoidosis, and as described in the clinical case, ACE values ​​may be normal despite the diagnosis of sarcoidosis, but they can be elevated in up to 60% of patients [3,8].

Despite its low prevalence, cardiac sarcoidosis should always be considered in patients diagnosed with sarcoidosis, especially when presenting with suggestive symptoms such as chest pain, palpitations, syncope, cardiac contractility changes, and/or arrhythmias [9]. Cardiac sarcoidosis represents the second leading cause of mortality among individuals with sarcoidosis, affecting approximately 5-10% of patients [4,5]. Given the increasing prevalence, current guidelines advocate for screening for cardiac involvement in patients with sarcoidosis and symptoms, with MRI or PET, as was done in the described case [5]. Granulomatous involvement in cardiac sarcoidosis can occur in any cardiac region, with a predilection for the left ventricle, interventricular septum, and nodum [5,8]. The predominant clinical manifestations arise from conduction disturbances, manifesting as arrhythmias and, less frequently, as signs and symptoms of heart failure [5,9]. Supraventricular arrhythmias, especially atrial fibrillation, occur in approximately 32% of cases [5,8].

Diagnosing cardiac sarcoidosis is often challenging. Common tests like electrocardiograms and transthoracic echocardiograms typically lack the necessary sensitivity and specificity [9]. Cardiac involvement can be definitively diagnosed based on findings from cardiac MRI or fluorodeoxyglucose (FDG) PET-CT [3,5]. In most cases, imaging and other findings (systemic) are sufficient to establish the diagnosis, with no need for myocardial biopsy [3,5]. Several potential risk factors for developing cardiac sarcoidosis have been identified, including male sex, pre-existing electrocardiographic abnormalities, elevated serum N-terminal pro-B-type natriuretic peptide (NT-proBNP) levels, multi-organ involvement, and progressive pulmonary radiological findings [5].

While spontaneous remission is common, systemic therapy is recommended for patients with cardiac, neurological, or severe pulmonary involvement, or those experiencing constitutional symptoms [3]. Therapeutic interventions for sarcoidosis primarily focus on alleviating symptoms and preserving organ function. Current strategies aim to modulate the underlying inflammatory response, thus inhibiting granulomatous lesion formation and progression [10]. Corticosteroids play a critical role as the primary treatment for sarcoidosis. However, there's a lack of comprehensive data to guide the best start time, length, and amount for their use [2,3]. The recommended initial regimen includes prednisolone at 20-40 mg/day for three months, followed by gradual dose tapering. For patients resistant to corticosteroids, methotrexate at 10-15 mg/week can be used as second-line therapy, with anti-tumor necrosis factor (anti-TNF) agents like infliximab and adalimumab considered as third-line options. Higher corticosteroid doses do not alter disease progression, but combination therapy with corticosteroid-sparing agents is often necessary [3]. Commonly used agents include methotrexate, azathioprine, leflunomide, and mycophenolate [3,11]. Hydroxychloroquine is particularly effective for cutaneous manifestations, certain cases of nervous system involvement, and hypercalcemia [12]. In cases of cardiac sarcoidosis, anti-TNF therapies may offer therapeutic advantages [10].

Cardiac sarcoidosis treatment involves pharmacological treatments and device-based therapies, with steroids and pacemakers showing good results. Pharmacological treatments include methotrexate and glucocorticoid, or glucocorticoid, and reassessment with PET/CT at month three to decide further treatment. Advanced heart block patients need pacemakers, and those with ventricular arrhythmias often require an implantable cardioverter-defibrillator (ICD) [5,8]. In our case, the option of ICD implantation was not considered due to the absence of clinical improvement with corticosteroid therapy.

Active disease does not necessarily imply a progressive course, a fatal prognosis, or even the need for treatment [1,3]. Most patients with sarcoidosis are asymptomatic or present with acute symptoms that resolve spontaneously; approximately one-third of patients experience a chronic course, which can progress inexorably if left untreated [8]. The presence of cardiac, pulmonary, neurological, and/or renal involvement is associated with higher morbidity and mortality [8]. Sudden cardiac death is considered the major cause of death in cardiac sarcoidosis [3,5].

## Conclusions

Sarcoidosis, a diagnosis of exclusion, is identified by clinical presentation and non-necrotizing granulomas in histology. This disease, although uncommon, should be considered in the differential diagnosis of patients with complaints such as chest pain or dyspnea, associated with systemic inflammation.

This case demonstrates the high level of clinical suspicion required in patients diagnosed with sarcoidosis who present with atypical symptoms, particularly symptoms such as chest pain. In such cases, it is crucial to consider cardiac sarcoidosis as a differential diagnosis. The authors emphasize the importance of early diagnosis and treatment with corticosteroid therapy, especially in cardiac sarcoidosis, as it is crucial for recovery and prevention of future complications.
